# Endogenous neurotoxin-like protein Ly6H inhibits alpha7 nicotinic acetylcholine receptor currents at the plasma membrane

**DOI:** 10.1038/s41598-020-68947-7

**Published:** 2020-07-20

**Authors:** Yasuhiro Moriwaki, Natsuki Kubo, Mizuho Watanabe, Shinsuke Asano, Tomoki Shinoda, Taro Sugino, Daiju Ichikawa, Shoutaro Tsuji, Fusao Kato, Hidemi Misawa

**Affiliations:** 1 Division of Pharmacology, Faculty of Pharmacy, Keio University, Tokyo, 105-8512 Japan; 2 Division of Clinical Physiology and Therapeutics, Faculty of Pharmacy, Keio University, Tokyo, 105-8512 Japan; 3 Molecular Diagnostics Project, Kanagawa Cancer Center Research Institute, Yokohama, 241-8515 Japan; 4 Department of Neuroscience, Jikei University School of Medicine, Tokyo, 105-8461 Japan

**Keywords:** Ion channels in the nervous system, Molecular neuroscience

## Abstract

α7 nicotinic acetylcholine receptors (nAChRs) are widely expressed in the central nervous system and regarded as potential therapeutic targets for neurodegenerative conditions, such as Alzheimer’s disease and schizophrenia. Yet, despite the assumed pathophysiological importance of the α7 nAChR, molecular physiological characterization remains poorly advanced because α7 nAChR cannot be properly folded and sorted to the plasma membranes in most mammalian cell lines, thus preventing the analyses in heterologous expression system. Recently, ER-resident membrane protein NACHO was discovered as a strong chaperone for the functional expression of α7 nAChR in non-permissive cells. Ly6H, a brain-enriched GPI-anchored neurotoxin-like protein, was reported as a novel modulator regulating intracellular trafficking of α7 nAChR. In this study, we established cell lines that stably and robustly express surface α7 nAChR by introducing α7 nAChR, Ric-3, and NACHO cDNA into HEK293 cells (Triple α7 nAChR/RIC-3/NACHO cells; TARO cells), and re-evaluated the function of Ly6H. We report here that Ly6H binds with α7 nAChRs on the cell membrane and modulates the channel activity without affecting intracellular trafficking of α7 nAChR.

## Introduction

Neuronal nicotinic acetylcholine receptors (nAChRs) are pentameric ligand-gated cation channels composed of combinations of eight α (α2-7, α9, α10) and three β (β2-4) subunits^1,2^. Among those, heteromeric α4β2 and homomeric α7 nAChR subtypes are the two major types abundantly expressed in the central nervous system^3^. High expression levels of α7 nAChR in the hippocampus, cerebral cortex, and several subcortical limbic regions suggest its contribution to higher brain functions, such as cognition, attention, memory, and sensory-gating ^2^, while impaired α7 nAChR signalling has been implicated in cognitive deficits associated with Alzheimer’s disease and schizophrenia^1,4,5^. In addition, the expression of α7 nAChRs has been detected in various non-neuronal cells, including immune cells, where it plays a role in immunity and inflammation^6,7^. Thus, α7 nAChR modulation has emerged as a novel therapeutic strategy for several neurologic and inflammatory disorders^4,5^.

α7 nAChRs retain several crucial characteristics that make the subtype peculiar, such as faster desensitisation kinetics and higher Ca^2+^ permeability compared to other nAChRs^8,9^. In addition, α7 nAChRs can be activated by choline, and are blocked by α-bungarotoxin (α-Bgtx) and methyllycaconitine (MLA), whereas α4β2 nAChRs are insensitive to choline, and are not affected by α-Bgtx and MLA^10–12^. In addition to fast desensitization kinetics, α7 nAChRs are neither properly oligomerised nor functionally activated in most cell lines, except a few neuronal or neuroendocrine cell lines after transfection with α7 nAChR cDNA^13–16^. To overcome these limitations, α7 and glycine chimeric receptor (α7-GlyR) was designed and used to study the ligand-binding domain of α7 nAChR^17,18^.

It is well known that in most non-permissive cells, the efficiencies in folding/assembly of α7 nAChR are very poor, and only a small portion of the α7 nAChR subunit is correctly incorporated into an active pentameric receptor in the plasma membrane^19^. The recent extensive research activities on α7 nAChR folding/cell surface expression have identified several ER-resident α7 nAChR chaperones^20^. Resistance to inhibitors of cholinesterase 3 (Ric-3) was first identified in *Caenorhabditis elegans* as a positive effector of nAChR maturation^21^. RIC-3 reportedly enhances the function of certain mammalian nAChRs, including α7 nAChR^22–24^, whereas conflicting effects of RIC-3 on α7 nAChR folding/maturation have also been reported^15,16^. Furthermore, in mice, endogenous Ric-3 is not expressed in the hippocampal dentate gyrus neurons that are rich in α7 nAChRs^22^. These observations suggest mammalian Ric-3 was neither necessary nor sufficient for efficient assembly of mammalian α7 nAChRs. Recently, a potent ER-resident membrane protein NACHO was discovered as a robust α7 nAChR chaperone^25^.

Potent α7 nAChR inhibitors, α-Bgtx and α-cobratoxin, which have been well-characterised, retain a unique three-finger structure (TFS) that is essential for their binding with α7 nAChR^26,27^. In mammals, there are several TFS-containing proteins belonging to the Ly-6/neurotoxin superfamily (Ly6SF), some of which are anchored by glycosylphosphatidylinositol (GPI), and have been reported to modulate nAChRs^28,29^. Of these, the most characterised Ly6SF protein Lynx1 has been shown as “a cholinergic brake” to accelerate desensitisation and reduce the sensitivity of α7 nAChR to its agonist^30^, and to modulate α4β2 subunit oligomerisation^31^. Recently, another member of GPI-anchored Ly6SF protein, Ly6H, was reported as a novel α7 nAChR negative modulator, where it suppresses intracellular trafficking of α7 nAChR^32^.

In this study, we established a cell line, which stably and robustly express surface α7 nAChRs by introducing α7 nAChR, Ric-3, and NACHO cDNAs into HEK293 cells (Triple α7 nAChR/RIC-3/NACHO cells; TARO cells), and analyzed the function of Ly6H onto the surface α7 nAChRs.

## Results

### Establishment of TARO (Triple α7 nAChR/RIC-3/NACHO) cells which stably express functional α7 nAChR in the plasma membrane

**Figure 1:**
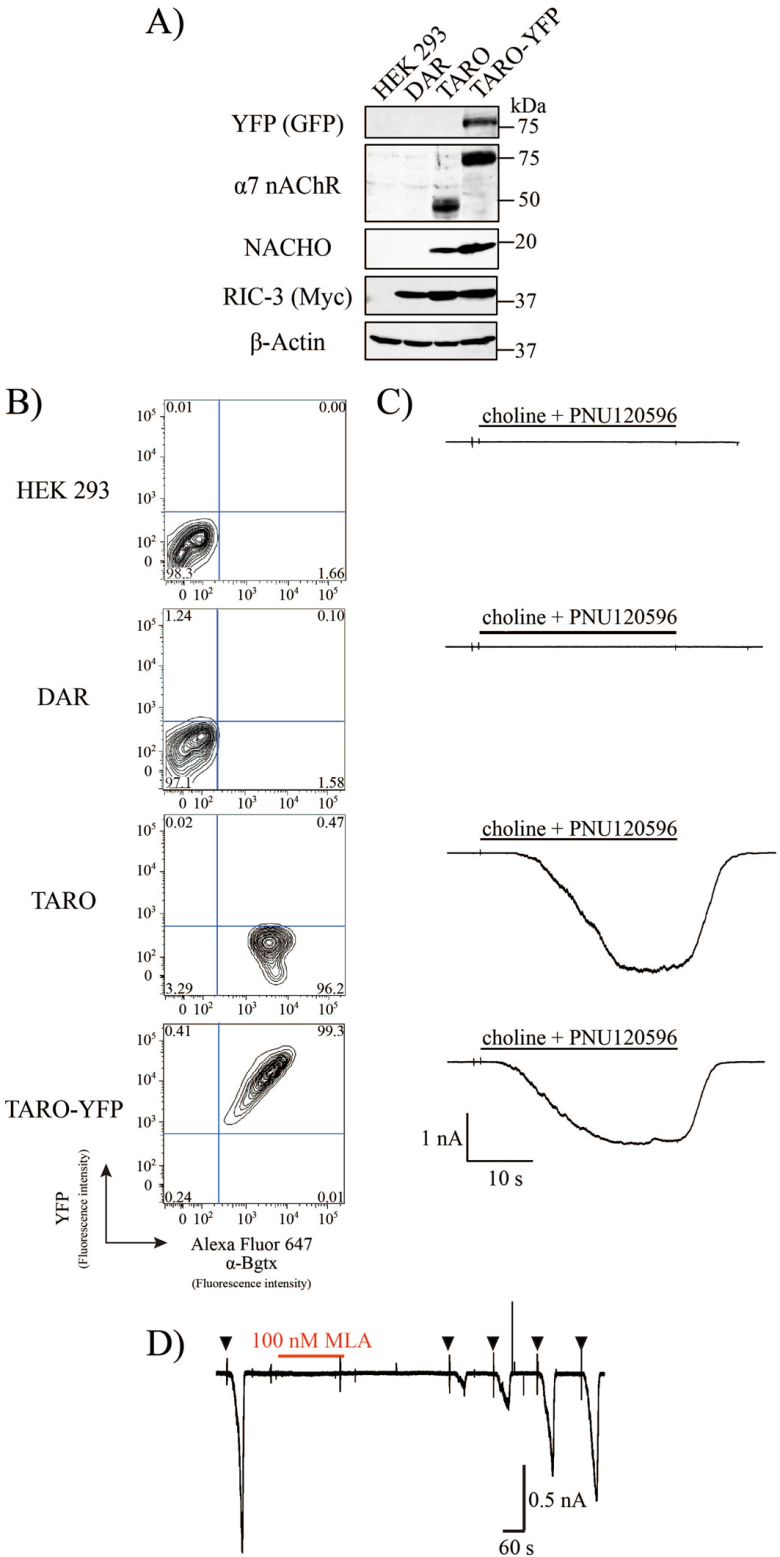
Establishment of a TARO cell, functionally active α7 nAChR stably expressing cell. (**A**) Representative immunoblots of indicated cells using anti-GFP, anti-Nicotinic Acetylcholine Receptor (α7 Subunit), anti-TMEM35 (NACHO), anti-Myc or anti-β-Actin antibody. (**B**) Cell surface expression of α7 nAChR was examined using flow cytometry. Cells were treated with Alexa Fluor 647-conjugated α-Bgtx. (**C**) Representative whole-cell recordings from indicated cells using α7 nAChR-selective agonist choline (30 μM), together with α7 nAChR positive allosteric modulator PNU120596 (1 μM). (**D**) Consecutive response of TARO cell to 40 s challenges with 30 μM choline and 1 μM PNU120596 (arrows) before and after applied for 3 min with 0.1 μM of α7 nAChR-selective agonist MLA.

While expression of α7 nAChR in HEK293 cells was limited when it was co-expressed with RIC-3 (Fig. 1A, lane 2), the expression level of α7 nAChR or YFP-tagged α7 nAChR was much higher when NACHO was also added (Fig. 1A, lane 3, 4). We termed the former cell lines with double expression of nAChR and RIC-3 “DAR” cells and the latter with triple expression of α7 nAChR, RIC-3 and NACHO “TARO” cells. Treatment of TARO with AlexaFluor 647-conjugated α-Bgtx, a snake venom toxin binding to properly-folded α7 nAChRs^8,11,33^, revealed that this increased expression of α7 nAChRs in TARO is accompanied by its increased surface expression in agreement with results by Gu et al. (Fig. 1B)^25^. In TARO cells, application of 30 μM choline^10^ in the presence of a positive allosteric modulator of α7 nAChRs, PNU120596^34^ resulted in a large inward current (> 1 nA; Fig. 1C) sensitive to MLA (100 nA; Fig. 1D), indicating these surface α7 nAChRs were functional. Choline-induced current was not detectable in naïve HEK293 and DAR cells. Hereafter we used TARO cells as cell line stably expressing functional α7 nAChRs.

### Ly6H interacts with and inhibits choline-evoked current of α7 nAChR

**Figure 2:**
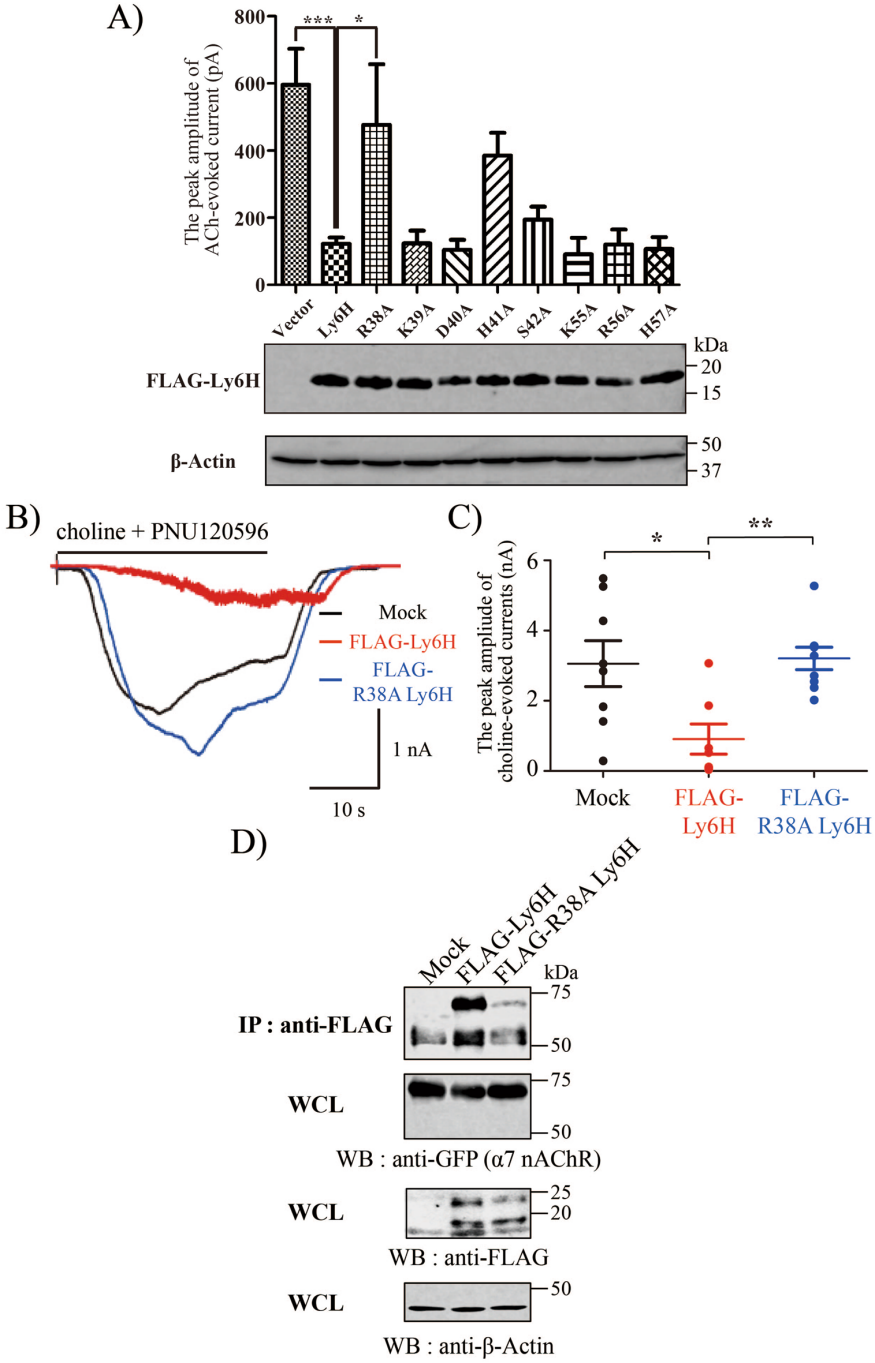
R38A mutant Ly6H abrogates the suppressive effect on choline-evoked currents of α7 nAChR. (**A**) HEK293 cells were transfected with pCI-gCHRNA7-hGLRA1 and pCherry/P2A or pCherry/P2A FLAG-Ly6H or pCherry/P2A FLAG-Ly6H mutants, and whole-cell ACh-evoked currents were recorded. Bar graphs represent the means ± SEM of peak currents (n = 3–20; *P < 0.05, ***P < 0.001, compared with Ly6H value, one-way ANOVA with Dunnett’s post hoc tests). The lower panel shows the expression profiles of Ly6H and Ly6H mutant proteins. (**B**) Representative whole-cell recordings from indicated TARO cells stimulated with 30 μM choline together with 1 μM PNU120596. (**C**) Quantification of choline and PNU120596-evoked peak currents in (**B**). Student’s *t* test *P* values : Mock vs Ly6H = *P < 0.05, Ly6H vs R38A Ly6H = **P < 0.01. (**D**) Representative immunoblots of FLAG-tagged Ly6 proteins co-immunoprecipitated with YFP-tagged α7 nAChR in TARO-YFP. Top, Immunoprecipitation with anti-FLAG followed by western blotting with anti-GFP. Upper middle, 5% input whole cell lysates (WCL) western blotting with anti-GFP. Lower middle, 5% input WCL western blotting with anti-FLAG. Bottom, 5% input WCL western blotting with anti-β-Actin as loading control.

We firstly investigated the suppressible effect of Ly6H on acetylcholine (ACh)-evoked current of α7 nAChR using the α7-GlyR chimera that was used as a rational model for evaluating the α7 nAChR function^17,18,32^. In the assay using α7-GlyR, we found that Ly6H is a functional negative modulator for ACh-evoked current (Fig. 2A). Next, to find out the critical residues of Ly6H on inhibiting ACh-evoked current of α7-GlyR, we made several types of Ly6H alanine mutants (Supplementary Fig. 1). The previous structural studies identified important residues within the TFS of α-Bgtx, α-Cobratoxin, and Lynx1 for binding with α7 nAChR located in the loop II of TFS^18,26,27^. According with these findings, R38A mutant which located in the loop II within the TFS of Ly6H showed the greatest effect; it completely abrogated the suppressive effect on α7-GlyR (Fig. 2A). Next, we used TARO cells to verify the effect of R38A mutant of Ly6H to native α7 nAChR. As with α7-GlyR expressed cells, Ly6H (Fig. 2A), but not R38A mutant, substantially suppressed the choline-evoked α7 nAChR currents in TARO cells (Fig. 2B, C).

To further investigate whether the Ly6H or R38A mutant proteins bind to α7 nAChRs, we evaluated the interaction of these proteins using TARO-YFP cells using immunoprecipitation. As shown in Fig. 2D, α7 nAChR was co-immunoprecipitated when Ly6H was pulled down. In contrast, R38A mutant lost the binding ability to α7 nAChR (Fig. 2D). These results indicate that Ly6H interacts with α7 nAChR through the R38 residue, located in the loop II within the TFS of Ly6H, and this interaction is required for inhibiting α7 nAChR currents.

### Ly6H suppresses α7 nAChR activity on the plasma membrane

**Figure 3:**
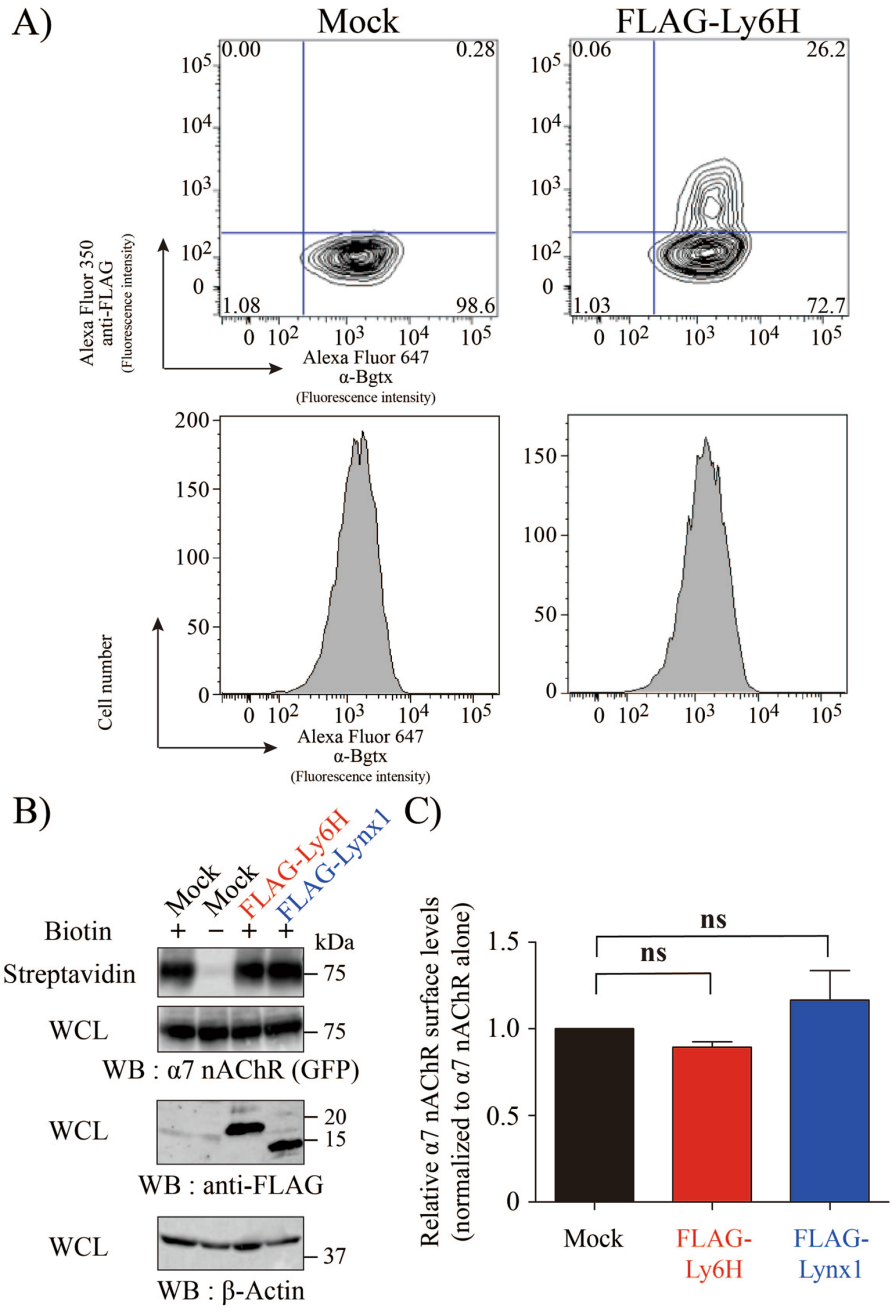
Ly6H is not involved in α7 nAChR trafficking in the presence and absence of NACHO. (**A**) Cell surface expression of α7 nAChR was examined using flow cytometry. TARO cells transfected with pCherry/P2A or pCherry/P2A FLAG-Ly6H were treated with anti-FLAG antibody followed by Alexa Fluor 350-conjugated antibody and Alexa Fluor 647-conjugated α-Bgtx. Lower left quadrant : cells lacking both Ly6H and α-Bgtx expression, Upper left quadrant : cells expressing Ly6H but not reacted with α-Bgtx, Lower right quadrant : cells lacking Ly6H expression but reacted with α-Bgtx, Upper right quadrant : cells expressing Ly6H and reacted with α-Bgtx. (**B**) Representative immunoblots of streptavidin-precipitated surface biotinylated α7 nAChR in TARO-YFP cells transiently transfected with pCherry/P2A, pCherry/P2A FLAG-Ly6H, or pCherry/P2A FLAG-Lynx1. Top, streptavidin-precipitation followed by western blotting with anti-GFP. Upper middle, 5% input WCL western blotting with anti-GFP. Lower middle, 5% input WCL western blotting with anti-FLAG. Bottom, 5% input WCL western blotting with anti-β-Actin as loading control. (**C**) The amount of biotinylated α7 nAChR and total α7 nAChR expression, in Top panel and Upper middle panel of (**B**) respectively, were quantified using the Image J software. The results shown are means ± SEM of the ratio of α7 nAChR cell surface expression over the α7 nAChR whole cell expression normalised by Mock transfection (n = 3).

Puddifoot et al. reported that Ly6H reduces cell-surface expression of α7 nAChR by inhibiting receptor trafficking from the ER to the plasma membrane^32^. In this study, we analysed whether Ly6H could affect α7 nAChR trafficking using TARO cells. TARO cells were transfected with Mock or FLAG-Ly6H, and were labelled with Alexa Fluor 647-conjugated α-Bgtx and anti-FLAG antibody, followed by Alexa Fluor 350-conjugated secondary antibody. Flow cytometry analyses revealed that Ly6H transfection (Fig. 3A upper right quadrant) did not change cell-surface α-Bgtx binding as compare with mock or non-transfected cells (Fig. 3A lower right quadrant). Next, we used a biotinylation assay to evaluate the effect of Ly6H on α7 nAChR intracellular trafficking. When we normalized the ratio of α7 nAChR cell surface expression over the α7 nAChR whole cell expression, no significant difference was observed between the Ly6H-expressing cells and Mock cells (Fig. 3B, C).

**Figure 4:**
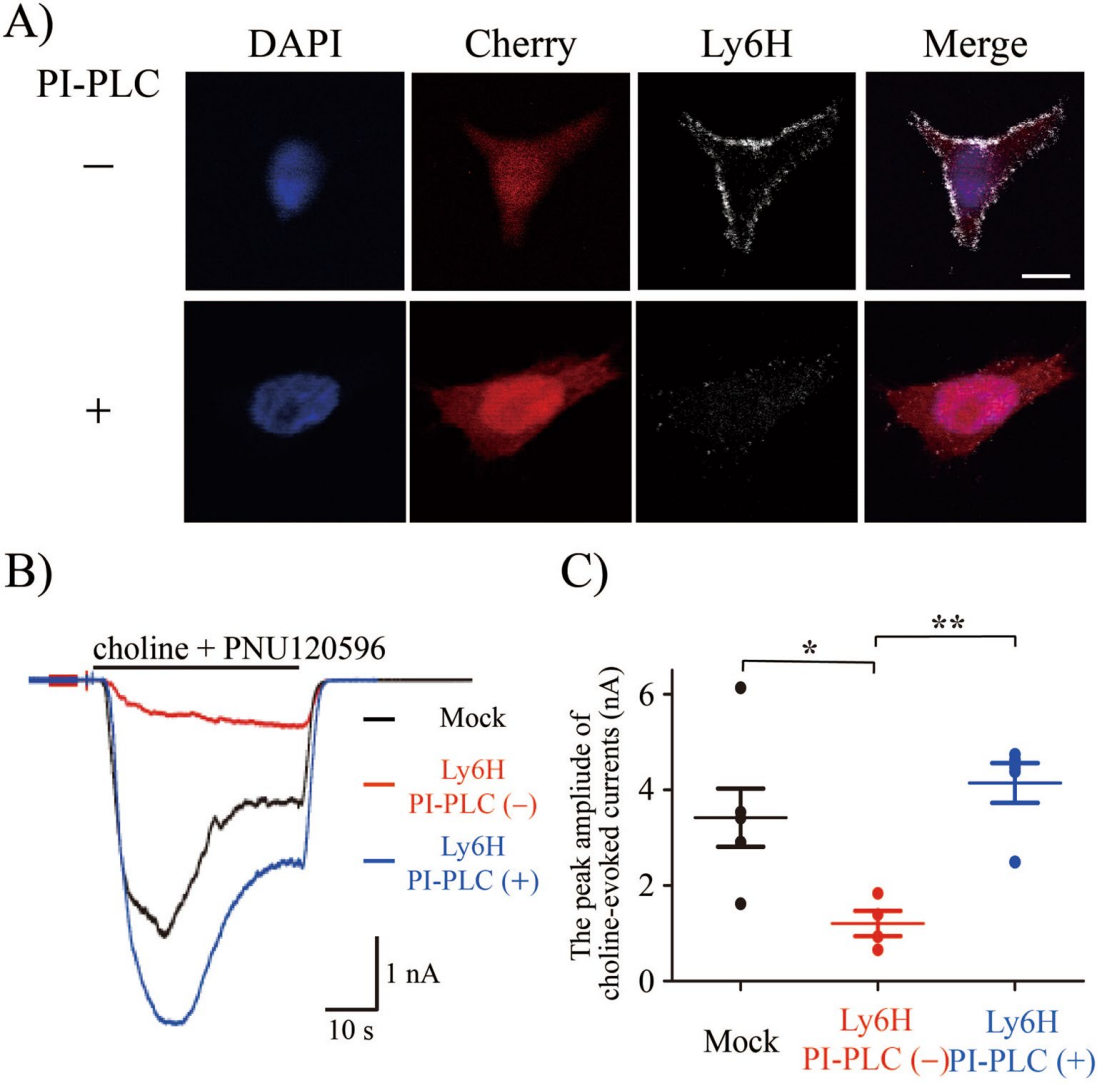
PI-PLC treatment completely abrogated Ly6H suppressible effect on choline-evoked currents in TARO cells. (**A**) Representative confocal microscopic images of DAPI (blue) for nuclear staining, Cherry (red) for confirming transfection efficiencies, and Ly6H (white) staining in TARO cells treated with or without PI-PLC. Scale bar, 10 μm. (**B**) Representative whole-cell recordings from indicated cells stimulated with 30 μM choline together with 1 μM PNU120596. (**C**) Quantification of choline and PNU120596-evoked peak currents with or without PI-PLC treatment. Student’s *t* test *P* values : Mock vs non-treatment = *P < 0.05, non-treatment versus PI-PLC treated = **P < 0.01.

Ly6H is a GPI-anchored protein known to be tethered to the outside of the cell membrane. Incubating cells with PI-PLC can cut off GPI-anchor and release the protein moiety from the cell surface^31^. After transfecting Ly6H cDNA into TARO cells, the cells were treated with PI-PLC. Because, Ly6H cDNA was connected with Cherry cDNA via 2A peptide cDNA, parallel expression of Ly6H proteins and Cherry proteins were observed (Fig. 4A upper panel). Cell surface Ly6H signals, which were clearly visible on the cell membrane, were completely abolished by the PI-PLC treatment (Fig. 4A lower panel). Next, we analysed choline-evoked α7 nAChR currents by the patch clamp configuration. The Ly6H-induced inhibition of α7 nAChR activity was abolished by the PI-PLC treatment (Fig. 4B, C).

**Figure 5:**
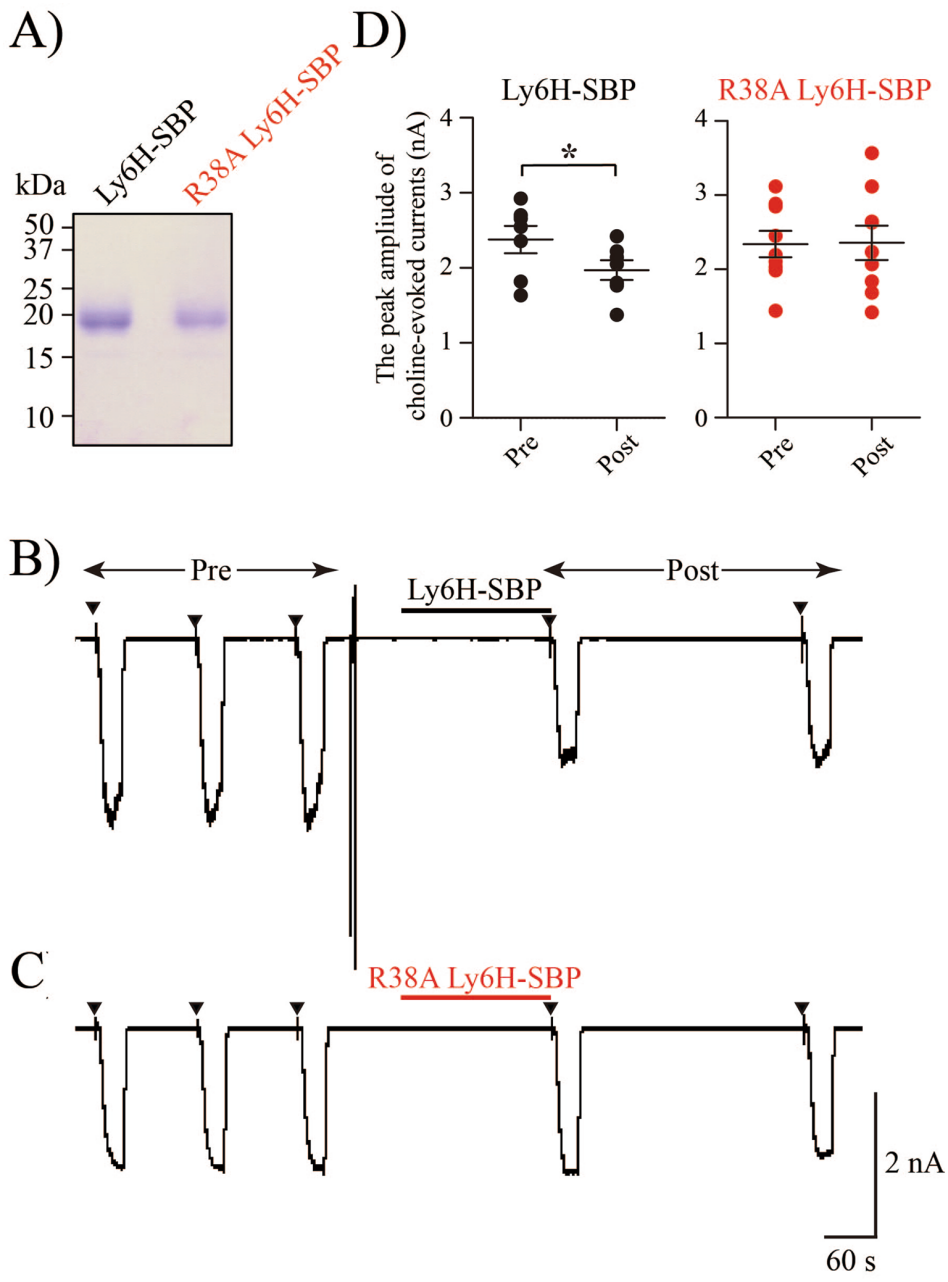
Soluble Ly6H protein suppresses choline-evoked currents in TARO cells. (**A**) CBB-stained bands of purified Ly6H-SBP and R38A Ly6H-SBP proteins. Consecutive response of TARO cells to 30 s challenges with 30 μM choline and 1 μM PNU120596 (arrows) before and after, applied for 3 min with 2.5 μM of (**B**) soluble Ly6H-SBP or (**C**) soluble R38A Ly6H-SBP. (**D**) Inhibitory ratio was calculated by dividing post-stimulation peak amplitude with pre-stimulation peak amplitude (Ly6H-SBP: n = 8 82.7%, R38A Ly6H-SBP: n = 9 100%). *P*-value is determined using a *t*-test. *P < 0.05.

Finally, we tried to assess whether an extracellularly added soluble Ly6H protein affects TARO cell responses to choline. Soluble streptavidin-binding peptide (SBP)-tagged Ly6H or Ly6H R38A mutant proteins were purified (Fig. 5A). Three-minute applications of 2.5 μM Ly6H-SBP, but not R38A Ly6H-SBP, decreased the choline-induced α7 nAChR currents (Fig. 5B, C). Mean amplitudes before and after treatment with Ly6H-SBP and R38A Ly6H-SBP were Ly6H-SBP; 2,377.5 ± 443.3 pA (before) and 1968.5 ± 322.5 pA (after), R38A Ly6H-SBP; 2,339.4 ± 503.2 pA (before) and 2,356.9 ± 657.7 pA (after). Small but significant reduction by Ly6H-SBP was detected compared with by R38A Ly6H-SBP (*p* < 0.05) (Fig. 5D). Overall, these results suggest that Ly6H does not affect α7 nAChR trafficking in the presence of NACHO, but suppresses the ligand-evoked α7 nAChR currents acting from outside the cells.

## Discussion

In this study, stable cell lines, which express three cDNAs each coding for α7 nAChR, Ric-3, and NACHO (TARO), or α7 nAChR-YFP, Ric-3, and NACHO (TARO-YFP) were established. Higher amounts of α7 nAChR surface expression and robust ligand-evoked α7 nAChR currents were detected in the cells. We then analysed the effect of Ly6H on the intracellular trafficking and ligand-evoked channel activity of α7 nAChRs in the cells. Until the discovery of NACHO by Gu et al.^25^, functional α7 nAChRs could not be expressed in almost all cell lines, except a few neuronal or neuroendocrine cell lines^13–16^. In the present study using TARO or TARO-YFP cells, we found that Ly6H binds with α7 nAChRs at the plasma membrane and inhibits the ligand-evoked channel activity without affecting intracellular trafficking of α7 nAChR.

During the course of the study, we created DAR (expressing α7 nAChR and Ric-3 without NACHO) and TARO cells. In DAR cells, we found the lower/negligible expression levels of α7 nAChR compared with TARO cells even though the same expression levels of RIC-3 were observed (Fig. 1A). Gu et al. showed that NACHO knockout mice completely lacked the functional expression of α7 nAChR in the brain^25^. Koperniak et al. showed complete knockdown of Ric-3, which was shown to be essential for nAChR responses in *C. elegans*, did not diminish α7 nAChR function in rat pituitary cells^15^. These results indicate NACHO is not only an essential chaperone protein responsible for the functional expression of α7 nAChRs in the plasma membrane, but also is critical factor for maintaining the stability of α7 nAChR protein. In order to substantiate these observations, HEK293 cells were transiently transfected with cDNAs coding for α7 nAChR + Ric-3, α7 nAChR + NACHO or α7 nAChR + NACHO + Ric-3, and α7 nAChR surface expression (α-Bgtx binding) was evaluated by FACS analyses (Supplementary Fig. 2). The cell surface α7 nAChR signals were more robust in α7 nAChR + NACHO + RIC-3 cells compared with α7 nAChR + NACHO cells, confirming that RIC-3 has some positive effects on α7 nAChR assembly/membrane expression at least in HEK293 cells, although RIC-3 alone has a limited effect on α7 nAChR surface expression.

Previously Puddifoot et al*.* transiently transfected YFP-tagged α7 nAChR, Ric-3, and myc-tagged Ly6H cDNAs into HEK293 cells, and analysed the cell surface α7 nAChR expression using the biotinylation assay^32^. They found that Ly6H co-expression reduced the cell surface α7 nAChR expression by ca. 50%. In the present study, we analysed the effects of Ly6H using TARO cells, in which functional α7 nAChRs were stably and robustly expressed. We first evaluated whether Ly6H can suppress ion channel activity of α7 nAChR by a patch clamp method. In accordance with a previous report by Puddifoot et al.^32^, suppressive effects of Ly6H on ion channel activity of α7 nAChRs were observed (Fig. 2). We also detected the suppressive effect of Ly6H in the cells using the α7-GlyR chimeric channel, which contained the extracellular ligand binding domain of α7 nAChRs, confirming that the Ly6H site of action is extracellular. We further identified the essential amino acids within the TFSs of Ly6H. Like other neurotoxins and Ly6SF members^18,26,27^, R38 of Ly6H is located in loop II of TFS, which is reported as a necessary region for binding to nAChR. Our FACS and biotinylation analyses indicated that Ly6H expression did not change the α7 nAChR surface expression under the condition of NACHO-assisted high-surface expression of α7 nAChRs (Fig. 3). These results demonstrate Ly6H binds to extracellular region of α7 nAChR at the plasma membrane through loop II region and inhibits the ligand-evoked channel activity without affecting intracellular trafficking of α7 nAChR. Because R36 of α-Bgtx and R33 of α-cobratoxin in their respective loop II regions of TFS are reported to bind directly with Y184 of α7 nAChR^26,27^, Ly6H may also directly bind to the same region of α7 nAChR through R38 in the loop II region.

The suppressive effect of Ly6H in the plasma membranes was also supported by the following findings. The PI-PLC treatment, which digested GPI anchor and released the tethered protein moieties from GPI-anchored proteins, cancelled the effect of Ly6H (Fig. 4). Next we treated TARO cells with a soluble Ly6H protein (Ly6H-SBP) and found a significant reduction in ligand-evoked α7 nAChR currents (Fig. 5). Inhibition by exogenously-added soluble Ly6H-SBP (14%) was low compared with the effect by co-expressing GPI-anchored Ly6H (70%). It could be possible that a high local concentration is required or binding affinity of Ly6H to α7 nAChR is relatively low. These ideas are also supported by our findings that Ly6H expressing TARO cell gradually showed reactivity to choline (30 μM) plus PNU-120596 (1 μM) (Figs. 2B, 4B). In this regard, it is interesting to note that Lyukmanova et al*.* used 10 μM of soluble Lynx1, applied for 1.5 min, and obtained a 10% reduction of acetylcholine-evoked currents compared to pre-treated cells^18^. In our condition, dosage of LY6H-SBP was calculated to be 2.5 μM. Stoichiometric binding analyses of Ly6H and α7 nAChR present an important avenue for future research.

Ly6H is highly expressed in the brain and immune cells and its expression is regulated in developmental stages^35,36^. Although the precise localisation of Ly6H at each cell population is unknown, we speculate that Ly6H may fine-tune the cell surface α7 AChR activity in time- and cell-specific manners. Besides the typical ligand-gated ion channel, α7 nAChRs are known to transmit various intracellular signals responsible for neuroprotection and anti-inflammation^4,5^. It will be a subject of future study whether Ly6H could also act as a signalling modifier in α7 nAChR-expressing neuronal or non-neuronal cells in physiological and pathological conditions.

## Materials and methods

### Plasmid construction

A DNA fragment encoding human Ric-3 [NM_024557] in frame with Myc-tag and P2A peptide^37^ was synthesised by GeneArt Gene Synthesis (Thermo Fisher Scientific, Inc., Rockford, IL, USA) and inserted into AgeI- and XhoI-digested (in this region DS-Red Monomer was coded) pDS-Red Monomer C1 vector (Clontech, Mountain View, CA, USA) using the In-Fusion HD Cloning Kit (Clontech), yielding the pRic-3 Myc/P2A vector. The gene encoding human CHRNA7 with YFP fused into M3-M4 loop was obtained from Addgene (Plasmid #62630; Watertown, MA, USA). Human CHRNA7 or CHRNA7 with YFP was then inserted into XhoI- and EcoRI-digested pRic-3 Myc/P2A vector, in frame, at the back of Ric-3 Myc/P2A using In-Fusion HD Cloning Kit.

The cDNA encoding Cherry protein was PCR-amplified by 5′-**CGCTAGCGCTACCGGT**CGCCACCATGGTGAGCAAGGGCGAG-3′ with 15 bp pDS-Red Monomer C1 homologous sequence (bold) and 5′-**GAAGCTTGAGCTCGAGAGGTCCAGGGTTCTCCTCCACGTCTCCAGCCTGCTTCAGCAGGCTGAAGTTAGTAGCTCCGCTTCC**CTTGTACAGCTCGTCCAT-3′ with P2A sequence (underline), and 15 bp pDS-Red Monomer C1 homologous sequence (bold). The PCR product was then inserted into AgeI- and XhoI-digested pDS-Red Monomer C1 vector using the In-Fusion HD Cloning Kit, yielding the pCherry/P2A vector. The DNA fragments encoding human Lynx1 [NM_177457] and mouse Ly6H [NM_011837] with N-terminal FLAG-tag were synthesised by GeneArt Gene Synthesis and inserted into XhoI- and BamHI-digested pCherry/P2A vector, in frame, at the back of Cherry/P2A using In-Fusion HD Cloning Kit. Several Ly6H mutants were generated using PCR-based mutagenesis strategies and were confirmed by dsDNA sequencing.

A DNA fragment encoding chicken CHRNA7 external region [NM_204181 : 1–681] and human GLRA1 cytosolic region [NM_000171 : 736–1347] in frame with HA-tag was synthesised by GeneArt Gene Synthesis and inserted into EcoRI-digested pCI-neo vector (Promega K.K., Tokyo, Japan), using In-Fusion HD Cloning Kit, yielding the pCI-gCHRNA7-hGLRA1 vector.

A synthetic DNA fragment encoding human NACHO (TMEM35) [NM_021637] was inserted into the EcoRI- and XhoI-digested pcDNA3.1 vector (Thermo Fisher Scientific, Inc.) using the In-Fusion HD Cloning Kit.

### Cell culture and transfection

Human embryonic kidney (HEK) 293 (RCB2202) and RK13 cells were cultured in Dulbecco’s modified Eagle’s medium (DMEM) supplemented with 10% foetal bovine serum (FBS), 100 U/mL of penicillin, and 100 μg/mL streptomycin at 37 °C in a 5% CO_2_/95% air atmosphere on culture dishes or cover glass. HEK293 cells were transfected using lipofectamine LTX and Plus reagent (Thermo Fisher Scientific, Inc.) according to the manufacturer’s instructions. To select stable transformants, G418 (1.0 mg/mL) was also added to the medium.

### Whole-cell patch-clamp

TARO cells were transfected with 2.5 μg pCherry/P2A FLAG-Ly6H. Three hours later, the cells were plated on 13-mm glass coverslips placed in 35-mm cell culture dishes. Twenty-four hours later, glass coverslips were transferred to dishes on the microscope stage. Protein expression was visually monitored under an upright microscope (BX-50WI and BX-51WI; Olympus, Tokyo, Japan). Whole-cell voltage clamp recordings were made from transfected TARO cells using an Axopatch 200B amplifier (Molecular Devices, Sunnyvale, CA, USA). Membrane currents were recorded at a holding potential of -50 mV (corrected for liquid junction potential). Microelectrodes were filled with internal solution composed (in mM) of 110 CsCl_2_, 10 HEPES, 5 BAPTA 4 K, 2 Magnesium ATP, 1 CaCl_2_ (pH 7.3, adjusted with NaOH), with resistances in the range of 3–5 megaohms. The bath was continuously perfused with extracellular solution containing (in mM) 140 NaCl_2_, 5 KCl, 10 glucose, 10 HEPES, 1 MgCl_2_, and 2 CaCl_2_ (pH 7.3, adjusted with NaOH). Atropine (1 μM) was included in the extracellular solution to block muscarinic ACh receptors. α7 nAChR-selective agonist choline (30 μM), together with α7 nAChR positive allosteric modulator PNU120596 (1 μM) was delivered using a 3-barrel square glass connected to a Perfusion Fast-Step (Warner instruments, Hamden, CT, USA). Agonists were applied for 40 s, which was triggered by the pCLAMP 10 software.

### Immunoprecipitation and western blot

The cells were homogenised in lysis buffer (50 mM Tris–HCl [pH 7.4], 150 mM NaCl, 10% glycerol, 1% Triton X-100 and 2% n-Octyl-β-D-Glucopyranoside) supplemented with Protease Inhibitor Cocktail (Nacalai Tesque, Kyoto, Japan). Extracts were clarified by centrifugation and protein concentrations were determined by the BCA protein assay. For immunoprecipitation, cell lysates were incubated with mouse anti-FLAG M2 monoclonal antibody (Merck Millipore, Billerica, MA, USA), followed by captured with Protein G Magnetic Beads (New England Biolabs, Beverly, MA, USA). Cell lysates and immunoprecipitated samples were subjected to SDS-PAGE. Thereafter, proteins were transferred to polyvinylidene difluoride (PVDF) membranes (Immobilon-P; Merck Millipore) and were probed with specific primary antibodies, and then with the appropriate HRP-conjugated secondary antibodies (Bio-Rad Laboratories, Inc., Hercules, CA, USA). Immuno-positive proteins were detected using the ECL Western Blotting Detection Reagent (GE Healthcare, Madison, WI, USA). For loading control, the membrane was probed with a monoclonal antibody against β-actin (MAB1501, 1:1,000 dilution; Merck Millipore). The antibodies used in this study were as follows: monoclonal anti-FLAG M2 antibody (1:500 dilution), monoclonal anti-GFP antibody (GF200, 1:1,000 dilution; Nacalai Tesque), polyclonal anti-Myc antibody (1:1,000 dilution; MBL, Woburn, MA, USA), monoclonal anti-Nicotinic Acetylcholine Receptor (α7 Subunit) antibody (306, 1:1,000 dilution; Merck Millipore), polyclonal anti-TMEM35 antibody (1:1,000 dilution; Merck Millipore), and monoclonal anti-SBP antibody (1:1,000 dilution; Merck Millipore).

### Flow cytometry

For detection of α7 nAChR or FLAG-Ly6H expressed on cell surface, cells were stained using Alexa Fluor 647-conjugated α-Bgtx (Merck Millipore) or anti-FLAG M2 antibody following incubation with Alexa Fluor 350-conjugated anti-mouse antibody (Thermo Fisher Scientific, Inc.). Stained cells were examined using flow cytometry with an LSR II Flow Cytometer running FACSDiva Software v8.0 (BD Biosciences, Franklin Lakes, NJ, USA). A total of 10,000 events were collected from each sample.

### Cell-surface biotinylation

Surface proteins on TARO cells were biotinylated using 1.2 mg/mL EZ-Link sulfo-NHS-SS-biotin and quenched as described previously ^38^. Labelled cells were rinsed once then lysed in 0.4 mL RIPA buffer with complete protease inhibitors, and 500 μg biotinylated proteins were precipitated using Neutr-Avidin Plus UltraLink Resin (Thermo Fisher Scientific, Inc.). The precipitates were analysed using western blotting.

### Immunocytochemistry

TARO cells transfected with pCheryy/P2A FLAG-Ly6H in cover glass were treated with or without 0.35 U/mL phosphatidylinositol-specific phospholipase C (PI-PLC; Thermo Fisher Scientific, Inc.) in OPTI-MEM (Thermo Fisher Scientific, Inc.) for 1 h at 37 °C, and fixed for 15 min with 4% paraformaldehyde (PFA) in 0.1 M phosphate buffer at pH 7.4, then rinsed three times with PBS. Thereafter, cells were blocked with the blocking buffer (5% normal donkey serum (NDS) in PBS) for 30 min. Primary antibodies (monoclonal anti-FLAG M2 antibody, 1:100 dilution;) were applied in the blocking buffer for 1 h. After two washes in PBS, incubation (1 h) with donkey Alexa Fluor 647-conjugated anti-mouse antibody in blocking buffer was performed. After washing with PBS, cells were incubated with 4′, 6-diamidino-2-phenylindole dihydrochloride (DAPI) (Merck Millipore) to visualise nuclei, washed again and post-fixed with 2% PFA in 0.1 M PB for 10 min. PFA was removed by three rinses with PBS, coverslips were mounted in Fluoremount-G (SouthernBiotech, Birmingham, AL, USA), and examined using an Olympus FV-1000 confocal system with a 60 × objective lens (N.A. = 1.35).

### Purification of recombinant mouse Ly6H-SBP fusion proteins

A synthetic DNA fragment encoding mouse Ly6H, in which C-terminal glycophosphatidylinositol (GPI) anchor signal was replaced with SBP^39^, was inserted into NheI- and NotI-digested pEF1α-IRES vector (Clontech) using In-Fusion HD Cloning Kit, yielding pLy6H-SBP vector. R38A mutation was introduced into pLy6H-SBP vector using PCR-based mutagenesis strategies and confirmed using dsDNA sequencing. These plasmids were transfected into RK13 cells using Lipofectamine 2000 (Thermo Fisher Scientific, Inc.) and stable clones were generated by G418 selection at a concentration of 0.8 mg/mL for two weeks. Culture supernatants were collected form these stable transformants and recombinant Ly6H-SBP or Ly6H R38A-SBP proteins were purified using streptavidin sepharose (GE Healthcare). Protein concentrations were determined using a BCA Protein Assay kit and SDS-PAGE, and stained with Coomassie brilliant blue (CBB). Proteins (2.5 μM) in external solution were applied to TARO cells for 3 min (0.1 mL/min), then choline-evoked currents were examined.

### Statistical analysis

The data are presented as mean ± standard error of the mean (SEM), unless otherwise indicated. The results were analysed statistically using one-way analysis of variance (ANOVA) followed by Dunnett’s post hoc test. Otherwise, unpaired Student’s *t*-test was used to estimate differences between means. Data analyses were performed using GraphPad Prism 5.0 (GraphPad Software Inc., La Jolla, CA, USA). Differences were considered statistically significant when *P* values were less than 0.05.

## Supplementary information

Supplementary information
