# Supplementary material for: Endogenous neurotoxin-like protein Ly6H inhibits alpha7 nicotinic acetylcholine receptor currents at the plasma membrane
Source: Sci Rep. 2020 Jul 20;10:11996. doi: 10.1038/s41598-020-68947-7 (PMC7371702; doi:10.1038/s41598-020-68947-7)
Supplement: Supplementary file 1 — Supplementary information [file 41598_2020_68947_MOESM1_ESM.pdf]

Supplementary Information

Endogenous neurotoxin-like protein Ly6H inhibits alpha7 nicotinic acetylcholine receptor currents at the plasma membrane

Yasuhiro Moriwaki[1*], Natsuki Kubo[1], Mizuho Watanabe[1], Shinsuke Asano[1], Tomoki Shinoda[1], Taro Sugino[1], Daiju Ichikawa[2], Shoutaro Tsuji[3], Fusao Kato[4], Hidemi Misawa[1*]

[1]Division of Pharmacology, [2]Clinical Physiology and Therapeutics, Faculty of Pharmacy, Keio University, 1-5-30 Shibakoen, Minato-ku, Tokyo 105-8512, Japan

[3]Molecular Diagnostics Project, Kanagawa Cancer Center Research Institute, Yokohama, Kanagawa, Japan

[4]Department of Neuroscience, Jikei University School of Medicine, Tokyo, Japan

* Address correspondence to : Yasuhiro Moriwaki, Ph.D. and Hidemi Misawa, Ph.D.

Division of Pharmacology, Faculty of Pharmacy, Keio University, 1-5-30 Shibakoen, Minato-ku, Tokyo 105-8512, Japan

Phone: +81-3-5400-2675

Fax: +81-3-5400-2698

E-mail: moriwaki-ys@pha.keio.ac.jp and misawa-hd@pha.keio.ac.jp

```
                                       I              II                                     III
α-Bgtx, Bungarus multicinctus       IVCHTTATSPISAVTCPPGENLCYRKMWCDAFCSSRGKVVELGCAATC-PSKKPY----------------EEVTCCSTDKCNPHP-KQRPG
α-Cobratoxin, Naja kaouthia       --IRCFITPD--ITSKDCPNG-HVCYTKTWCDAFCSIRGKRVDLGCAATC-PTVKTG----------------VDIQCCSTDNCNPFPTRKRP-
Ly6H,  Mus musculus               WCQDCTLANSSHCAPKQCQPTDTVCASVRITDPSSSRKDHSVNKMCASSC-DFVKRHFFSDYLMGFINSGILKVDVDCCEKDLCNG--------
Lynx1, Homo sapiens               DCHVCAYNGDNCFNPMRCPAMVAYCMTTR---TYYTPTRMKVSKSCVPRCFETVYDG-----------YSKHASTTSCCQYDLCNG--------
                                      *         .  *      *       .  :     *.  *.. *                           **. * **

                                     I               II                                    III
Ly6H, Pan troglodytes             WCQDCTLTTNSSHCTPKQCQPSDTVCASVRITDPSSSRKDHSVNKMCASSCDFVKRHFFSDYLMGFINSGILKVDVDCCEKDLCNG
Ly6H, Homo sapiens                WCQDCTLTTNSSHCTPKQCQPSDTVCASVRITDPSSSRKDHSVNKMCASSCDFVKRHFFSDYLMGFINSGILKVDVDCCEKDLCNG
Ly6H, Canis familiaris            WCQDCTLTTNSSHCTPKQCQPSDTVCASVRITDPSSSRKDHSVNKMCASSCDFVKRHFFSDYLMGFINSGILKVDVDCCGGDLCNG
Ly6H, Rattus norvegicus           WCQDCTLAN-SSHCAPKQCQPTDTVCASVRITDPSSSRKDHSVNKMCASSCDFVKRHFFSDYLMGFINSGILKVDVDCCEKDLCNG
Ly6H, Mus musculus                WCQDCTLAN-SSHCAPKQCQPTDTVCASVRITDPSSSRKDHSVNKMCASSCDFVKRHFFSDYLMGFINSGILKVDVDCCEKDLCNG
                                  *******:. ****:******:*********************************************************  *****
```

Supplementary Fig. S1. ClustalW alignment of Ly6H with long chain α-neurotoxins (α-bungarotoxin, α-cobratoxin) and Lynx1

Amino acid alignments were performed using the ClustalW2 program (http://clustalw.ddbj.nig.ac.jp/index.php?lang=ja). Predicted signal sequences and C-terminal cleaved domains of Ly6H and Lynx1 were removed before analysis. Identical residues are marked by an asterisk. Strongly similar residues are marked by a colon, and weakly similar residues are marked by a single dot. The black brackets show the disposition of the disulphide bridges, and the cysteine residues are coloured in red. I – III designate the respective disulphide loops in the TFS of these proteins. The residues marked in blue reportedly participate in interactions with nAChRs [18, 26, 27]. The residues marked in green are critical residues for interaction with α7 nAChR, clarified in this study.

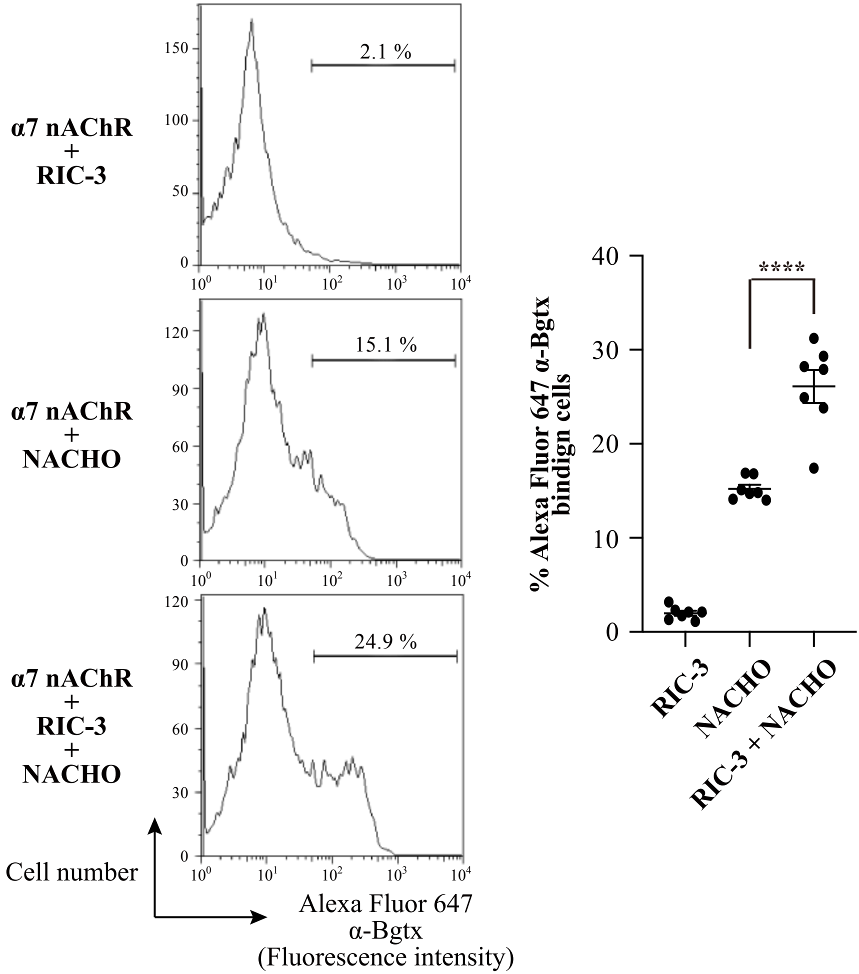

Supplementary Fig. S2. RIC-3 is not an essential but an auxiliary factor for α7 nAChR assemblly

Cell surface expression of α7 nAChR was examined using flow cytometry. HEK293 cells transiently transfected with α7 nAChR plus Ric-3, NACHO or Ric-3 & NACHO were treated with Alexa Fluor 647-conjugated α-Bgtx. Student's t test P values : NACHO vs RIC-3 + NACHO = ****$P<0.0001$.

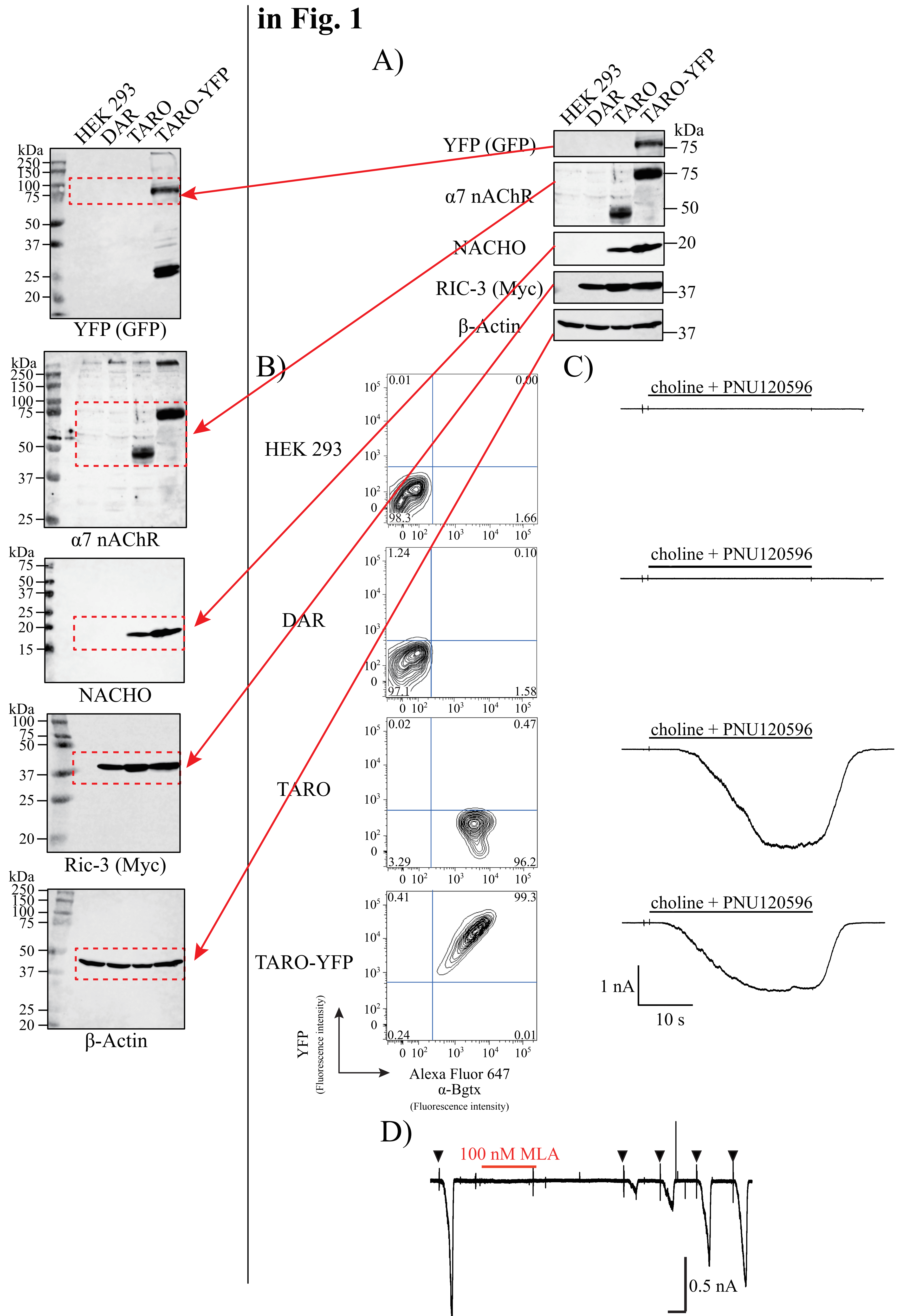

Supplementary Fig. S3. Full-length western blots of Figure 1.
Red dotted boxes show the cropping locations.

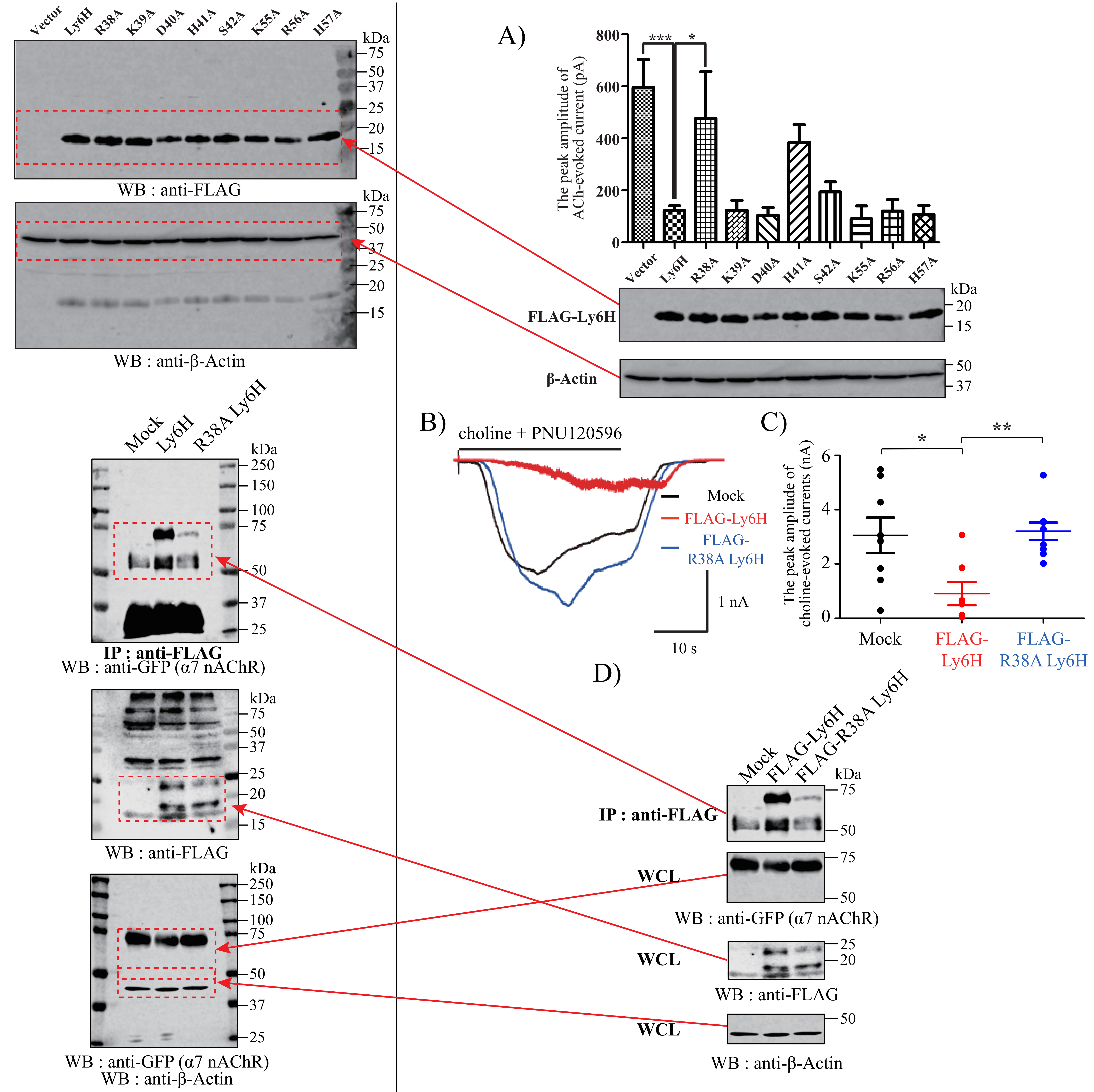

Supplementary Fig. S4. Full-length western blots of Figure 2.
Red dotted boxes show the cropping locations.

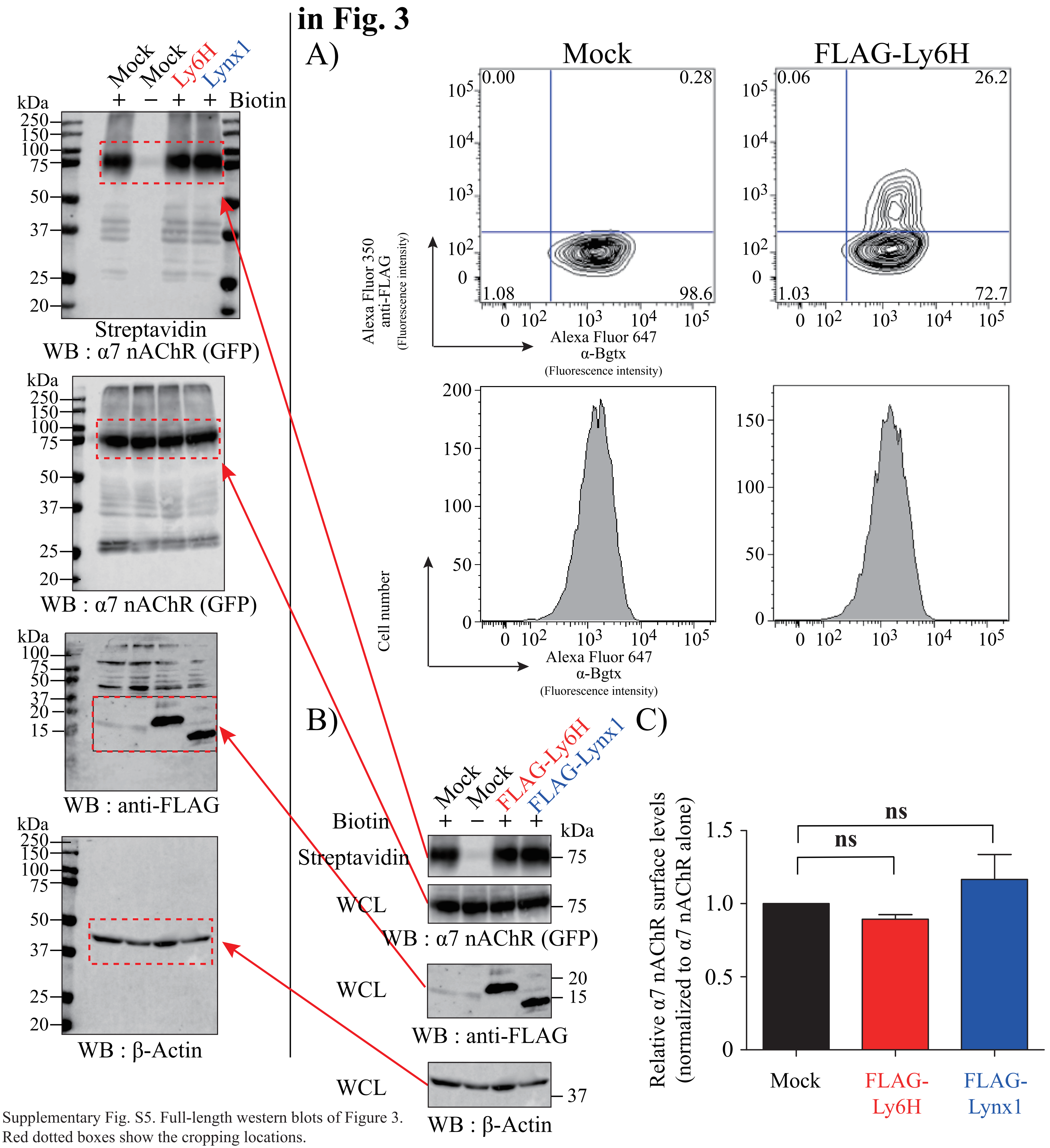

Supplementary Fig. S5. Full-length western blots of Figure 3.
Red dotted boxes show the cropping locations.
